# Residues 41V and/or 210D in the NP protein enhance polymerase activities and potential replication of novel influenza (H7N9) viruses at low temperature

**DOI:** 10.1186/s12985-015-0304-6

**Published:** 2015-05-05

**Authors:** Wenfei Zhu, Xiaohui Zou, Jianfang Zhou, Jing Tang, Yuelong Shu

**Affiliations:** National Institute for Viral Disease Control and Prevention, China CDC, Key Laboratory for Medical Virology, National Health and Family Planning Commission, 155 Changbai Road, Beijing, 102206 P.R China

**Keywords:** Influenza A (H7N9) virus, NP mutations, Low temperature replication

## Abstract

**Background:**

The influenza A (H7N9) virus emerged in the spring of 2013 in China. It contained six internal genes from Y280-like H9N2 viruses, which have co-circulated with G1-like lineage viruses throughout poultry in China. Accompanied with continuous reassortment among H7N9 and H9N2 viruses in poultry, it is possible for H7N9 viruses to acquire internal genes of G1-lineage viruses. Thus, it is important to evaluate potential impact of G1-like viruses on the H7N9 viruses.

**Findings:**

We used *in vitro* assays of polymerase activities and growth kinetics to evaluate the potential contribution of G1-like virus genes to the replication abilities of H7N9 viruses. Two mutations in the NP protein (41V and/or 210D) could enhance H7N9 RNP activities, especially at low temperature (33°C, which is similar to the temperature of human upper respiratory tract). Meanwhile, G1 viruses with V41I or D210E substitutions exhibited poor growth ability in the early infection stage at low temperature. The D210E substitution also reduced the replication ability of G1 virus at 12 and 24 hour post infection at 37°C. In both tested temperatures, V41I could compensate for the defective virus replication induced by the D210E mutation.

**Conclusions:**

Mutations 41V and/or 210D in the NP protein conferred improved RNP activity in H7N9 viruses and promoted the replication ability of H9N2 viruses, particularly at lower temperature. Substitutions at these two positions may promote the replication ability of H7N9 viruses in low temperature and thus might contribute to viral transmissibility. While these two residues have not yet been observed in H7N9 viruses, attention should be devoted to these two residues.

**Electronic supplementary material:**

The online version of this article (doi:10.1186/s12985-015-0304-6) contains supplementary material, which is available to authorized users.

## Findings

The zoonotic transmission of a novel influenza A (H7N9) virus into humans in February 2013 in Eastern China has become a global concern [1]. This H7N9 virus was a reassortant between H7 and N9 viruses, with six internal genes from Y280-lineage H9N2 avian influenza viruses. Although studies in avian and mammalian models such as chickens, pigs, ferrets, and non-human primates have shown that this virus is of low or mild pathogenicity [2-5], H7N9 infection in humans has often led to severe outcomes [1,6,7]. Glutamic acid (E)-to-lysine (K) substitution at position 627 in PB2, one of the important molecular markers of mammalian-adapted avian influenza viruses, was reported to be associated with enhanced virulence of the highly pathogenic H5N1 and H7N7 avian influenza viruses in mice [8,9], and responsible for viral replication at lower temperatures (33°C), allowing enhanced growth in the upper respiratory tract of mammals and efficient transmission of the viruses [10-13]. Although there has been limited human-to-human transmission [14], no highly transmissible cases have been reported for the novel H7N9 virus. This virus is reported to have more efficient replication in the lower respiratory tract than the upper respiratory tract [15], which merits studying the role of viral factors in transmissibility, especially viral replication at low temperature (33°C).

**Figure 1:**
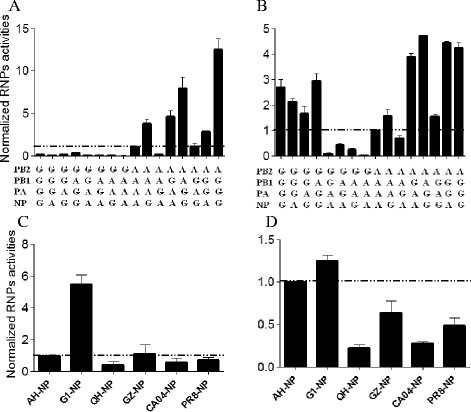
Normalized polymerase activities of recombinant polymerase complexes between A/Quail/Hongkong/G1/1997 (H9N2) and A/Anhui/1/2013 (H7N9) **(A & B)**, or different NPs in the backbone of A/Anhui/1/2013 (H7N9) PB2/PB1/PA plasmids **(C & D)**. RNP activities were measured in triplicate based on a Gluc reporter system and normalized to AH1 RNP activity at 33°C **(A, C)** and 37°C **(B, D)**. Abbreviations in **A** & **B**: G, A/Quail/Hongkong/G1/1997; A, A/Anhui/1/2013; Abbreviations in **C** & **D**: AH, A/Anhui/1/2013 (H7N9, AH1); G1, A/Quail/Hongkong/G1/1997 (H9N2, G1); QH, A/bar-headed goose/Qinghai/1/2005 (H5N1); GZ, A/Guangzhou/333/1999 (H9N2); CA04, A/California/04/2009 (2009pdmH1N1); and PR8, A/Puerto Rico/8/1934 (H1N1). Results are representative of three independent experiments.

Multiple genotypes of H7N9 viruses have been reported via continuous reassortant of their internal genes with H9N2 viruses in poultry [16]. Additionally, two different H9N2 lineages, Y280-like and G1-like, co-circulate in poultry in mainland China [17-19], raising the possibility for H7N9 viruses to reassort with G1-like virus. To test whether four RNP complex genes (PB2, PB1, PA, and NP) of G1-like H9N2 viruses confer any advantages to those of H7N9 viruses, we first tested all recombinant RNP activities between A/Quail/Hong Kong/G1/1997 (H9N2, G1) and A/Anhui/1/2013 (H7N9, AH1) at 33°C and 37°C. Temperatures of 33°C and 37°C were used to approximate the conditions of human upper and lower respiratory tracts, respectively. Reporter plasmid polI-Gluc [20] was co-transfected with expression plasmids encoding PB2, PB1, PA, and the NP of G1 or AH1 into 293T cells using the PolyFect (Qiagen, Valencia, CA, USA) reagent, according to the manufacturer’s instructions. Gluc activity in supernatants was analyzed in 24 hours post transfection using a Gluc assay kit (New England Biolabs, Beverly, MA, USA) and normalized to AH1 RNP activity. G1 exhibited about 2.5-fold higher RNP activity than AH1 at 37°C (Figure 1B), while at 33°C the RNP of G1 showed much less activity than AH1 (Figure 1A). PB2-E627K could have contributed to the better RNP function of AH1 at 33°C, because the G1 virus contained the 627E residue at PB2 protein. In addition, the AH1-NP led to decreased RNP activity of G1 at 37°C, while the G1-NP enhanced the RNP activity of AH1 at both temperatures (Figure 1A, B).

For a more detailed investigation of the role of different NPs in viral replication and transcription, we constructed four NP plasmids from different subtype viruses: A/bar-headed goose/Qinghai/1/2005 (H5N1, QH1), A/Guangzhou/333/1999 (H9N2, GZ), A/California/04/2009 (2009pdmH1N1, CA04), and A/Puerto Rico/8/1934 (H1N1, PR8). We co-transfected polI-Gluc with plasmids expressing the RdRp subunits of AH1 plus different NPs into 293T cells, and RNP activities were tested. G1-NP was the only plasmid that significantly enhanced RNP activity of AH1 at 33°C (Figure 1C), and also increased RNP activity at 37°C about 1.25 fold (Figure 1D).

**Figure 2:**
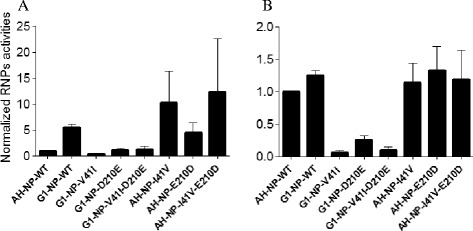
Viral RNA polymerase activities of NP-41I/41V and/or NP-210E/210D in 293T cells in the backbone of A/Anhui/1/2013 (H7N9). Luciferase-based minigenome reporter assays were used to measure polymerase activity in 293T cells at 33°C **(A)** or 37°C **(B)**. Cells were co-transfected with Gluc reporter plasmid and expression plasmids PB2, PB1, and PA of A/Anhui/1/2013 (H7N9), plus different NPs to generate different viral RNPs. After culturing at 33°C or 37°C for 24 h, *Gaussia* luciferase production was measured and normalized to AH1 activity. Results are presented as mean ± SEM and are representative of three independent experiments.

We then compared the sequences of the six tested NPs: AH1, G1, QH1, GZ, CA04, and PR8. G1-NP possessed two specific residues, 41V and 210D, while the other five NPs contained 41I and 210E (Additional file 1: Figure S1). To investigate whether the NP-41V and/or NP-210D mutations affect the enzymatic activity of RNPs, mutations NP-41V and/or NP-210D were introduced into the AH1-NP plasmid using a Site-Directed Mutagenesis Kit (Saibaisheng, Beijing, China). Conversely, mutations NP-41I and/or NP-210E were introduced into the G1-NP plasmid to generate mutant segments. The presence of the introduced mutation and the absence of additional unwanted mutations were verified by sequencing the entire plasmids. We then analyzed the activities of the recombinant RNP composed of AH1-PB2, AH1-PB1, AH1-PA, and various NPs (AH1-NP-WT, AH1-NP-I41V, AH1-NP-E210D, AH1-NP-I41V-E210D, G1-NP-WT, G1-NP-V41I, G1-NP-D210E, and G1-NP-V41I-D210E) in 293T cells at different temperatures using a mini-genome replication assay. Similar to the results exhibited in Figure 2, G1-NP-WT increased AH1-RNP activity at both 33°C and 37°C. Little effect was observed with G1-NP-V41I and/or G1-NP-D210E at 33°C, but reduced activity of V41I and D210E was shown at 37°C. Likewise, G1-NP with V41I and D210E decreased the G1-RNP-WT activities (Additional file 2: Figure S2). Moreover, AH1-NP with mutations I41V and/or E210D exhibited 5–20 fold increased RNP activity of AH1-WT at 33°C (Figure 2). These results indicate that residues 41V and 210D in NP proteins can enhance RNP activities of AH1 (H7N9), especially at 33°C.

**Figure 3:**
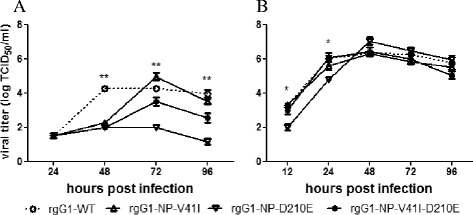
Replication kinetics of recombinant viruses rgG1-WT, rgG1-NP-V41I, rgG1-NP-D210E, and rgG1-NP-V41I-D210E in MDCK cells at 33°C **(A)** or 37°C **(B)**. Confluent monolayers of the various cell lines were inoculated with rgG1-WT, rgG1-NP-V41I, rgG1-NP-D210E, or rgG1-NP-V41I-D210E viruses. Cultured supernatants were harvested at 12, 24, 48, 72, and 96 hpi. Virus titers were determined by TCID_50_ assay using MDCK cells. Results are presented as mean ± SEM and are representative of three independent experiments. *, *p* < 0.05 and **, *p* < 0.001, compared the value of rgG1-NP-D210E with rgG1-WT.

To investigate the effect of NP-41V and/or 210D on virus replication, recombinant viruses were generated with reverse-genetics as described previously [21]. For biosafety concerns, the four rescued viruses were performed using G1 (H9N2) backbones: rgG1-WT, rgG1-NP-V41I, rgG1-D210E, and rgG1- NP-V41I-D210E. MDCK cells were infected with rescued viruses at a multiplicity of infection (MOI) of 0.001, and incubated in the appropriate medium containing 2 mg/L N-p-tosyl-L-phenylalaninechloromethyl ketone-treated (TPCK) trypsin (Sigma, Saint Louis, MO, USA) at 33 or 37°C. At 12, 24, 48, 72, and 96 hours post inoculation (hpi), supernatants were harvested and virus titers were determined using MDCK cells, as described previously [22]. As shown in Figure 3B, at 37°C, the D210E substitution in the NP protein significantly decreased the replication ability of rgG1-WT at early stages post infection (12 and 24 hpi (*p* < 0.05; *n* = 3), although all four viruses demonstrated comparable growth capability at later stages. Mutation NP-V41I alone or in combination with D210E, which showed decreased polymerase activity, exhibited little impact on the growth of rescued virus at 37°C. The apparent discrepancy between the minigenome reporter assay and the *in vitro* virus replication assay in our study may be related to the different parameters; whereas mRNA synthesis and reporter protein expression is the primary readout in minigenome assays, for efficient virus replication, a proper balance between the syntheses of cRNA, vRNA, and mRNA is required. However, at 33°C, G1 viruses with the D210E mutation displayed a replication defect (Figure 3A). The rgG1-WT virus demonstrated better replication ability to the other three viruses, except G1-NP-V41I of 72 hpi. These data indicate a growth advantage of recombinant viruses of rgG1-WT containing NP-41V and NP-210D in MDCK cells at 33°C, which corresponds to their high polymerase activity in 293T cells.

Sequence alignment of available NP sequences of avian and mammalian influenza A viruses in NCBI showed that in 14,142 NP sequences, 357 (2.5%) possess NP-41V and 156 (1.1%) possess NP-210D (Table 1). Most of the viruses with NP-41V or NP-210D residues were avian influenza viruses (197/357 and 143/156, respectively). However, few NP-41V or NP-210D residues were found in mammalian influenza viruses, including those of human and swine-origin viruses. In addition, no such substitutions have yet been found in the novel H7N9 viruses (data not shown). Despite this, our study revealed that compared with NP-210D, NP-210E could reduce the RNP activity of AH1 (H7N9) and G1 (H9N2) viruses, and could also inhibit the replication of G1 viruses, especially at 33°C. However, compared with NP-41V, although viruses with NP-41I were associated with decreased RNP activity, no significant variation in virus growth ability was observed. Therefore, mutations NP-41V and/or NP-210D may increase the growth capability of the AH1 (H7N9) virus, particularly in low temperature. Reassortants between G1-like and Y280-like viruses are not rare [17-19]. Given the continuous mutation and dynamic reassortment [16,23] of H7N9 viruses in both human and avian hosts, the possibility for these viruses to obtain these two residues or the entire NP gene of G1-like viruses cannot be excluded. Therefore, despite the absence of amino acids 41V and 201D in the NP of current H7N9 viruses, reassortants with the entire G1-like NP gene and substitutions of these two residues in the NP protein are worthy of attention.

**Table 1:** **Database search for NP-41I/V and/or NP-210D/E mutations in virus isolates from nature**

**Mutations**	**Human**	**Swine**	**Avian**	**Canine**	**Equine**	**Other residues**
NP-41V	6	14	197	42	98	I(13,763), A(2), G(3), K(1), M(1), Q(8), T(6), Y(1)
(n = 357)						
NP-210D	9	4	143	0	0	E(13960), G(2), N(9), P(3), Q(2), R(3), X(7)
(n = 156)						
NP-41V + 210D	6	0	19	0	0	–
(n = 25)						

The data shown are the number of sequences with identical amino acid composition (NP-41 and NP-210) of 14,142 influenza isolates in the NCBI database. The total number of each residue is shown in parentheses.

## Additional files

Additional file 1: Figure S1. Sequence alignment of the NP genes of six viruses: A/Anhui/1/2013 (H7N9, AH1), A/California/04/2009 (2009pdmH1N1, CA04), A/Puerto Rico/8/1934 (H1N1, PR8), A/Guangzhou/333/1999 (H9N2, GZ), A/bar-headed goose/Qinghai/1/2005 (H5N1, QH), and A/Quail/Hong Kong/G1/1997 (H9N2, G1). Additional file 2: Figure S2. Viral RNA polymerase activities of NP-41I/NP-41V and/or NP-210E/210D in the background of A/Quail/Hongkong/G1/1997 (H9N2). 293 T cells were co-transfected with *Gluc* reporter plasmid and expression plasmids PB2, PB1, and PA of A/Quail/Hongkong/G1/1997 (H9N2), plus G1 NP with different mutations. After culturing at 37°C for 24 h, *Gaussia* luciferase production was measured and normalized to G1 activity. Results are presented as mean ± SEM and are representative of three independent experiments.

## Abbreviations

Gluc: *Gaussia* luciferase
RNP: Ribonucleoprotein
RdRp: Influenza RNA-dependent RNA polymerase
PB2: Polymerase basic 2 protein
PB1: Polymerase basic 1 protein
PA: Polymerase acidic protein
NP: Nucleoprotein
p.i.: post-infection
MDCK: Madin-Darby canine kidney
