# Supplementary figures and images for: Residues 41V and/or 210D in the NP protein enhance polymerase activities and potential replication of novel influenza (H7N9) viruses at low temperature
Source: Virol J. 2015 May 5;12:71. doi: 10.1186/s12985-015-0304-6 (PMC4434832; doi:10.1186/s12985-015-0304-6)

Additional file 1: Figure S1

↓ I41V

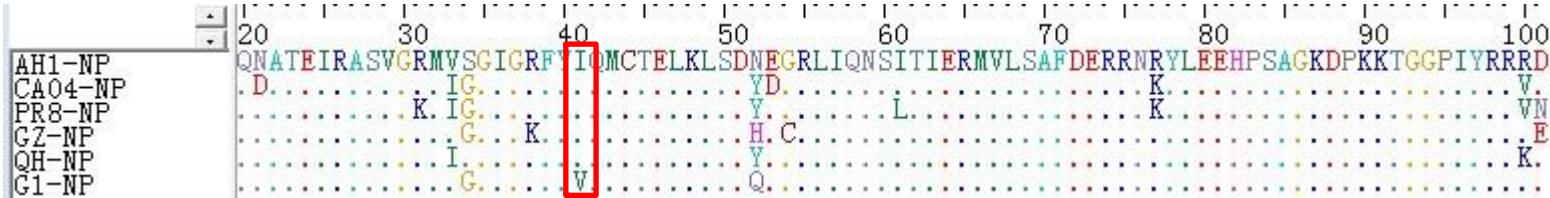

↑ E210D

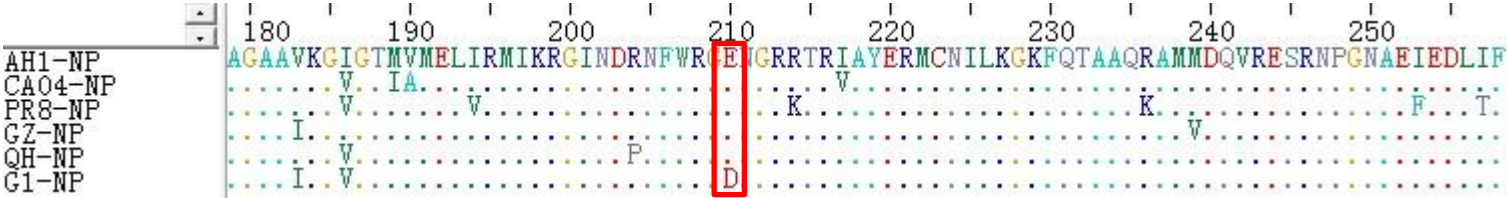

Supplement: Additional file 1: Figure S1. — Sequence alignment of the NP genes of six viruses: A/Anhui/1/2013 (H7N9, AH1), A/California/04/2009 (2009pdmH1N1, CA04), A/Puerto Rico/8/1934 (H1N1, PR8), A/Guangzhou/333/1999 (H9N2, GZ), A/bar-headed goose/Qinghai/1/2005 (H5N1, QH), and A/Quail/Hong Kong/G1/1997 (H9N2, G1). [file 12985_2015_304_MOESM1_ESM.pdf]

## Additional file 2: Figure S2

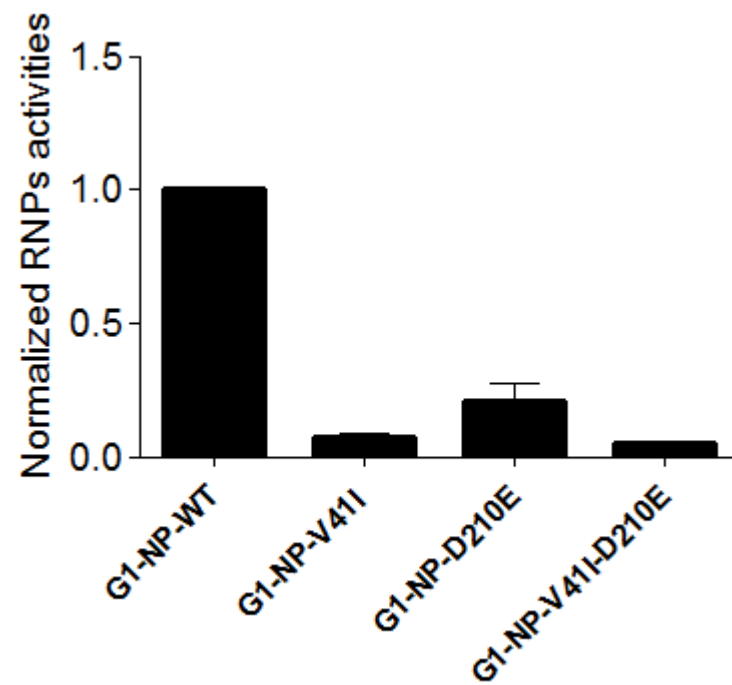

Supplement: Additional file 2: Figure S2. — Viral RNA polymerase activities of NP-41I/NP-41V and/or NP-210E/210D in the background of A/Quail/Hongkong/G1/1997 (H9N2). 293 T cells were co-transfected with Gluc reporter plasmid and expression plasmids PB2, PB1, and PA of A/Quail/Hongkong/G1/1997 (H9N2), plus G1 NP with different mutations. After culturing at 37°C for 24 h, Gaussia luciferase production was measured and normalized to G1 activity. Results are presented as mean ± SEM and are representative of three independent experiments. [file 12985_2015_304_MOESM2_ESM.pdf]
